# Study on the High-Temperature Reaction Kinetics of Solid Waste-Based High Belite Sulphoaluminate Cement Containing Residual Gypsum in Clinker

**DOI:** 10.3390/ma18143369

**Published:** 2025-07-17

**Authors:** Dunlei Su, Mingxin Yang, Yani Hao, Jiahui Wang, Xin Liu, Haojian Tang, Fengyuan Dong, Dejin Xing, Weiyi Kong

**Affiliations:** 1 School of Civil Engineering, Shandong Jiaotong University, Jinan 250357, China; sdl@sdjtu.edu.cn (D.S.); haoyani0118@163.com (Y.H.); wjh9542024@163.com (J.W.); yx_xgc@163.com (X.L.); thj9899@163.com (H.T.); 14753121209@163.com (F.D.); dejinxing@163.com (D.X.); 2 Shandong Key Laboratory of Technologies and Systems for Intelligent Construction Equipment, Jinan 250357, China

**Keywords:** high belite sulphoaluminate cement, solid waste, high-temperature reaction kinetics, activation energy, control mechanism

## Abstract

In order to elucidate the high-temperature reaction process of solid waste-based high belite sulphoaluminate cement containing residual gypsum in clinker (NHBSAC) and obtain the formation laws of each mineral in clinker, this article studied its high-temperature reaction kinetics. Through QXRD analysis and numerical fitting methods, the formation of C_4_A_3_$\bar{S}$, β-C_2_S, and CaSO_4_ in clinker under different calcination systems was quantitatively characterized, the corresponding high-temperature reaction kinetics models were established, and the reaction activation energies of each mineral were obtained. The results indicate that the content of C_4_A_3_$\bar{S}$ and β-C_2_S increases with the prolongation of holding time and the increase in calcination temperature, while CaSO_4_ is continuously consumed. Under the control mechanism of solid-state reaction, the formation and consumption of minerals follow the kinetic equation. C_4_A_3_$\bar{S}$ and β-C_2_S satisfy the D_4_ equation under diffusion mechanism control, and CaSO_4_ satisfies the R_3_ equation under interface chemical reaction mechanism control. The activation energy required for mineral formation varies with different temperature ranges. The activation energies required to form C_4_A_3_$\bar{S}$ at 1200–1225 °C, 1225–1275 °C, and 1275–1300 °C are 166.28 kJ/mol, 83.14 kJ/mol, and 36.58 kJ/mol, respectively. The activation energies required to form β-C_2_S at 1200–1225 °C and 1225–1300 °C are 374.13 kJ/mol and 66.51 kJ/mol, respectively. This study is beneficial for achieving flexible control of the mineral composition of NHBSAC clinker, providing a theoretical basis and practical experience for the preparation of low-carbon cement and the optimization design of its mineral composition.

## 1. Introduction

Cement is one of the basic raw materials widely used in the construction industry and plays an important role in social and economic development. Among them, Portland cement (OPC) and sulphoaluminate cement (SAC) are the most commonly used and technologically mature cement materials. However, the production process of OPC requires a large amount of mineral resources and causes certain environmental pollution, making the contradiction between energy, resources, and environment increasingly prominent [1,2,3,4]. Compared with OPC, the preparation of SAC significantly reduces the amount of limestone and CO_2_ emissions, but it requires substantial amounts of high-quality bauxite and gypsum resources [5,6,7]. In view of this, the research and development of low-carbon cement has become a hot topic [8,9].

High belite sulphoaluminate cement (HBSAC) is a low-carbon cement optimized from SAC, with a composition of 37–47% C_4_A_3_$\bar{S}$, 46–56% β-C_2_S, and 5–9% C_4_AF. Compared to SAC, its higher content of β-C_2_S further reduces the use of calcium, while its lower content of C_4_A_3_$\bar{S}$ reduces the use of aluminum and sulfur, allowing low-quality raw materials to be used for the preparation of HBSAC. At present, significant progress has been made in the study of HBSAC in the following areas: One is the feasibility study of using solid waste to prepare HBSAC. The solid waste materials that have been used to prepare HBSAC include aluminum silicate solid waste such as fly ash [10], tailings [11], coal gangue [12,13], and red mud [14], as well as calcium sulfur solid waste such as phosphogypsum [15], solid sulfur ash [16], and lithium mica slag [17]. The second is the study of the HBSAC calcination system. Under laboratory conditions, the calcination temperature of HBSAC is usually within the range of 1250–1350 °C, and the holding time is within the range of 20–60 min. Under industrial experimental conditions, the calcination system varies due to differences in production equipment and processes. The calcination temperature is generally around 1300 °C, while the holding time may differ due to differences in clinker production. During the study process, some experts and scholars have also reached some beneficial conclusions by improving the calcination system. Shen et al. [18] and Bullerjahn [19] successfully prepared HBSAC clinker containing C_5_S_2_$\bar{S}$ through the secondary calcination method. In this study, the “two-stage calcination” refers to reheating the clinker at 1100–1200 °C on the basis of the original calcination system. Li et al. [20] successfully prepared HBSAC using microwave sintering under a calcination system of 1150 °C for 10 min. The third aspect is the study of hydration properties. At present, the study conclusions on the hydration properties of HBSAC are basically consistent, with C_4_A_3_$\bar{S}$ hydration dominating in the early stage and β-C_2_S hydration dominating in the later stage. Due to the increased β-C_2_S content in the HBSAC clinker system, while ensuring rapid early strength development, it also solves the problem of slow later strength development in SAC.

In recent years, in order to understand the formation rules of HBSAC minerals, optimize their mineral composition, and ultimately improve their properties, experts and scholars have conducted in-depth exploration of HBSAC. Research has found that the high-temperature reaction processes of various minerals in clinkers often play a critical role in the final properties of clinkers. At present, research on mineral formation mainly adopts three methods. The first method is to qualitatively analyze the clinker minerals under different calcination systems to obtain their formation laws. Su et al. [11] used XRD analysis to obtain the formation rules of clinker minerals under different calcination systems and ultimately determined the temperature range for the formation of each mineral in the clinker. The second method is to analyze the BSE images of minerals under different calcination systems in order to understand the formation process of minerals at high temperatures. Liu et al. [21] studied the formation process and reaction mechanism of C_5_S_2_$\bar{S}$ by analyzing its BSE images under different calcination regimes. The results indicate that the formation process of C_5_S_2_$\bar{S}$ is roughly the reaction of SiO_2_ and CaO to generate CS (calcium silicate), which is further converted into C_2_S (dicalcium silicate) and combines with unconverted CS to form C_5_S_2_$\bar{S}$, and the entire process is controlled by a diffusion mechanism. The third method is to construct dynamic models of each mineral, analyze the kinetic parameters of each mineral, and then grasp its formation laws. In terms of study on C_4_A_3_$\bar{S}$, Ma et al. [22] successfully synthesized C_4_A_3_$\bar{S}$ single ore through the chemical reagent method and constructed its formation kinetics model. The results indicate that the formation process of C_4_A_3_$\bar{S}$ single ore is controlled by the interfacial chemical reaction mechanism, and the formation activation energy is 198.01 kJ/mol. It is worth noting that Li et al. [23] also obtained C_4_A_3_$\bar{S}$ single ore using the chemical reagent method but measured its formation activation energy to be 234 kJ/mol. Geng [24] successfully prepared C_4_A_3_$\bar{S}$ using solid waste as raw material. The results indicate that C_4_A_3_$\bar{S}$ is controlled by a diffusion mechanism and conforms to the Ginstling equation, with an activation energy of 85.63 kJ/mol. In terms of study on β-C_2_S, Xu et al. [25] investigated the high-temperature reaction kinetics of β-C_2_S, and the results showed that under the diffusion control mechanism, the formation process of β-C_2_S follows the D_4_ equation. This is consistent with the conclusion drawn by Hao [26] through numerical simulation to explore the reaction kinetics of β-C_2_S. In addition, Hao obtained an activation energy of 121.5 kJ/mol for β-C_2_S formation through simulation. However, current study results mainly focus on single minerals, and there is little study on the high-temperature reaction kinetics of multi-mineral synergistic systems in clinker.

Our team used industrial solid wastes such as petroleum coke ash (PCA), fly ash (FA), carbide slag (CS), and bauxite (BX) to prepare a high belite sulphoaluminate cement containing residual gypsum in clinker (NHBSAC). In order to further investigate the high-temperature reaction process of various minerals in clinker and reveal the formation laws of each mineral. We used methods such as QXRD analysis and numerical fitting to study the high-temperature reaction kinetics of various minerals in order to obtain the kinetic parameters of each mineral, clarify their formation laws under different calcination regimes, establish corresponding high-temperature reaction kinetics models, and provide a theoretical basis and practical experience for the preparation of low-carbon cement and the optimization design of its mineral composition.

## 2. Experimental

### 2.1. Experimental Materials

**Figure 1:**
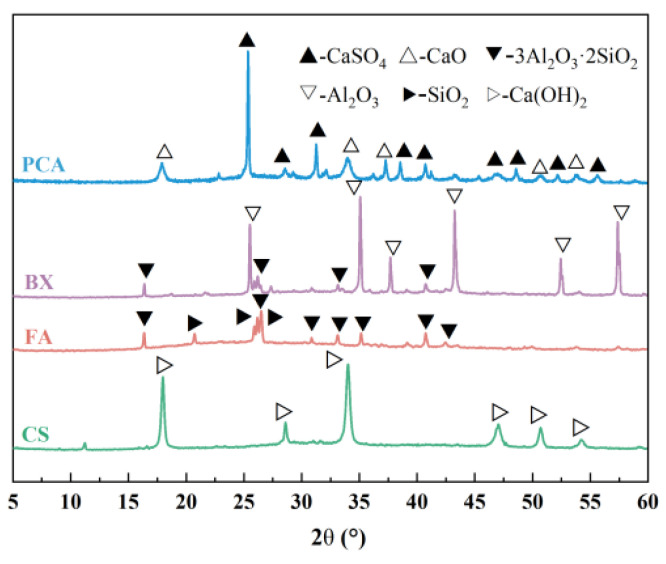
XRD patterns of raw materials.

NHBSAC was prepared from solid wastes such as PCA, FA, CS, and BX. Among them, PCA was purchased from Sinopec Qingdao Petrochemical Co., Ltd., Qingdao, China; FA was purchased from Henan Jinrun New Materials Co., Ltd., Zhengzhou, China; CS was purchased from Qingdao Haiwan Chemical Co., Ltd., Qingdao, China; and BX was purchased from Henan Borun Casting Materials Co., Ltd., Gongyi, China. The chemical composition and mineral composition of the aforesaid raw materials are shown in Table 1 and Figure 1.

### 2.2. Experimental Scheme

In order to reveal the reaction laws of clinker minerals at high temperatures, the high-temperature reaction kinetics mechanisms of each mineral were explored. In this study, a total of 20 experimental schemes were designed, with calcination temperatures set at 1200 °C, 1225 °C, 1250 °C, 1275 °C, and 1300 °C and holding times set at 15 min, 30 min, 45 min, and 60 min, respectively. Each scheme was obtained by combining different holding times and calcination temperatures. Table 2 shows the design of the experimental scheme and the proportion of raw materials.

### 2.3. Cement Preparation Process

**Figure 2:**
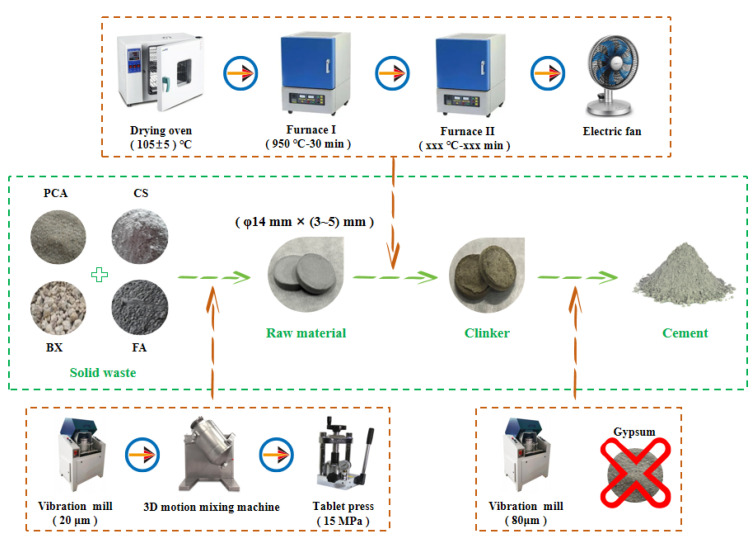
Preparation process of NHBSAC.

The preparation process of NHBSAC mainly follows the two grinding and one burning process, including grinding and molding, preheating and sintering, and cooling and regrinding, as shown in Figure 2. It is worth noting that this study adopted the “preheating and sintering” technology, which can not only more realistically simulate the industrial production process of “preheater → precalciner → rotary kiln” but also effectively improve the calcination efficiency of clinker.

### 2.4. Test Methods

(1) Loss on ignition: The determination method refers to the combustion difference method in the “Methods for Chemical Analysis of Cement” (GB/T 176-2008, China) [27].
(2) Chemical composition: A Model 1800 X-ray fluorescence spectrometer from Shimadzu, Kyoto, Japan, was utilized to analyze. The testing parameters included an Rh target X-ray tube (the voltage and current of the tube are 40 kV and 80 mA), a 4 kW thin window, and a scanning speed of 300°/min.
(3) Mineral composition: A D8 Advance X-ray diffractometer from Bruker, Karlsruhe, Germany, was utilized to analyze. The testing parameters included the following: a Cu target; Kα ray; voltage and current of the tube at 40 kV and 40 mA; qualitative and quantitative analyses with residence times of 0.05 s and 0.5 s; a scanning range of 5° to 60° for the 2θ angle; and a step width of 0.02°. The quantitative analysis of clinker minerals was conducted using FullProf 2020.6 software. The crystallographic data of the relevant minerals applied in Rietveld refinement are presented below: PDF#88-0812 for C_5_S_2_$\bar{S}$, PDF#71-0969 for C_4_A_3_$\bar{S}$, PDF#86-0398 for β-C_2_S, PDF#99-0010 for CaSO_4_, and PDF#77-0442 for TiO_2_.

## 3. Results and Discussion

### 3.1. Mineral Composition of Cement Clinker

#### 3.1.1. Qualitative Analysis

**Figure 3:**
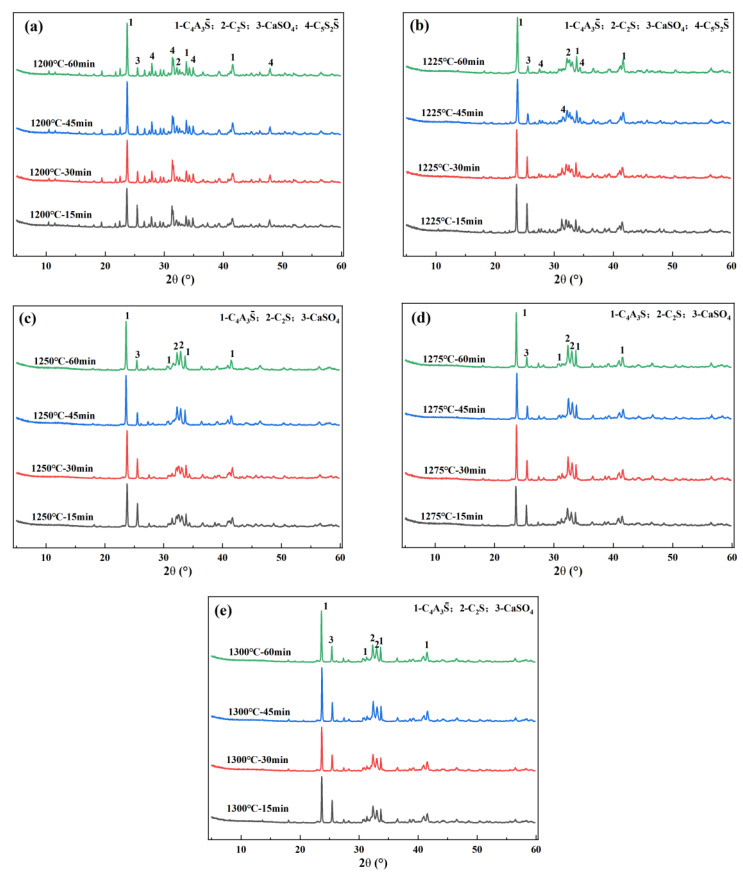
XRD analysis of clinkers under different calcination systems: (**a**) 1200 °C; (**b**) 1225 °C; (**c**) 1250 °C; (**d**) 1275 °C; and (**e**) 1300 °C.

According to the experimental schemes designed in Table 2, clinkers were calcined and then subjected to qualitative analysis. The results are shown in Figure 3. From this figure, it can be seen that the clinker under all conditions contains the target minerals C_4_A_3_$\bar{S}$, β-C_2_S, and CaSO_4_. Furthermore, as the calcination temperature rises and the holding time prolongs, the content of C_4_A_3_$\bar{S}$ and β-C_2_S continues to increase, and the content of CaSO_4_ continues to decrease. However, it should be noticed that when the temperature is not higher than 1225 °C, regardless of the holding time, intermediate mineral C_5_S_2_$\bar{S}$ will appear in the clinker minerals. When the temperature is higher than 1225 °C, C_5_S_2_$\bar{S}$ will disappear.

#### 3.1.2. Quantitative Analysis

**Figure 4:**
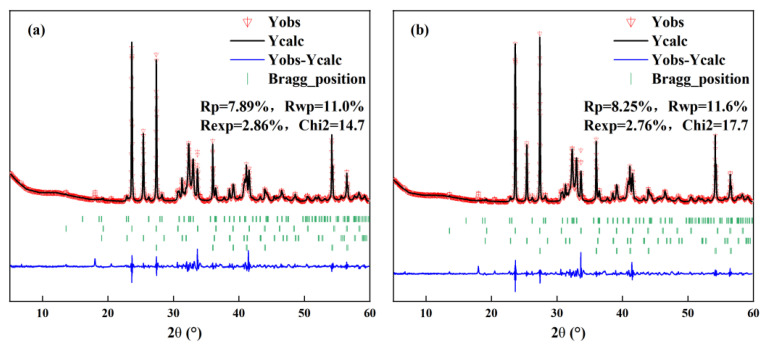
Analysis result of cement–TiO_2_ mixture under different calcination systems: (**a**) 1300 °C for 15 min and (**b**) 1300 °C for 30 min.

During the calcination process, amorphous phases will form in the clinker. Nevertheless, traditional crystal analysis techniques such as XRD are difficult to effectively characterize amorphous phases, which leads to deviations between the results of quantitative analysis and the actual values. In order to remove the impact of the amorphous phase, the internal standard method [28] was used for quantitative analysis. This method introduces a crystal with a known content that does not affect other minerals to assist analysis. In this study, TiO_2_ was adopted as the internal standard substance; its content in cement–TiO_2_ is 15%. The actual content of clinker minerals obtained through the internal standard method is shown in Table 3. The analysis results of the cement–TiO_2_ mixture under different calcination systems are shown in Figure 4. Due to limited space, only representative analysis results were presented.

### 3.2. High-Temperature Reaction Kinetics of C_4_A_3_$\bar{S}$

#### 3.2.1. Conversion Rate of C_4_A_3_$\bar{S}$

The conversion rate of mineral refers to the degree of mineral formation in clinkers; it can be described by the ratio of the mineral’s actual content to its designed content [29], as shown in Equation (1).

(1) $$\alpha =\frac{w_{n}}{W_{n}}\times 100\%$$

where *α*—conversion rate (%); *w_n_*—the actual content of clinker mineral (%); and *W_n_*—design content of clinker minerals (%).

As shown in Table 3, the content of C_4_A_3_$\bar{S}$ increases with the increase in calcination temperature and holding time. This is because the increase in calcination temperature and holding time is beneficial for promoting the reaction of CaSO_4_ with CaO and Al_2_O_3_, thereby accelerating the formation of C_4_A_3_$\bar{S}$. The formation process of C_4_A_3_$\bar{S}$ is shown in Equation (2).

(2) $$3CaO+3Al_{2}O_{3}+CaSO_{4}\to C_{4}A_{3}\bar{S}$$

In this study, the designed content of C_4_A_3_$\bar{S}$ is 35%. By using Equation (1) and the data in Table 3, the conversion rates of C_4_A_3_$\bar{S}$ under different calcination systems can be obtained, and the results are listed in Table 4.

#### 3.2.2. Kinetic Model of the C_4_A_3_$\bar{S}$ Formation

When preparing NHBSAC, the calcination temperature is relatively low and the liquid phase generated is less, so the high-temperature reaction is mainly a solid-state reaction. Table 5 shows several common control mechanisms and kinetic equations [30,31] of solid-state reactions. The kinetic equations in Table 5 can be represented by Equation (3).

(3) F(α)=Kt

where *t*—calcination time; *α*—reaction conversion rate (%); and *K*—reaction rate constant.

**Figure 5:**
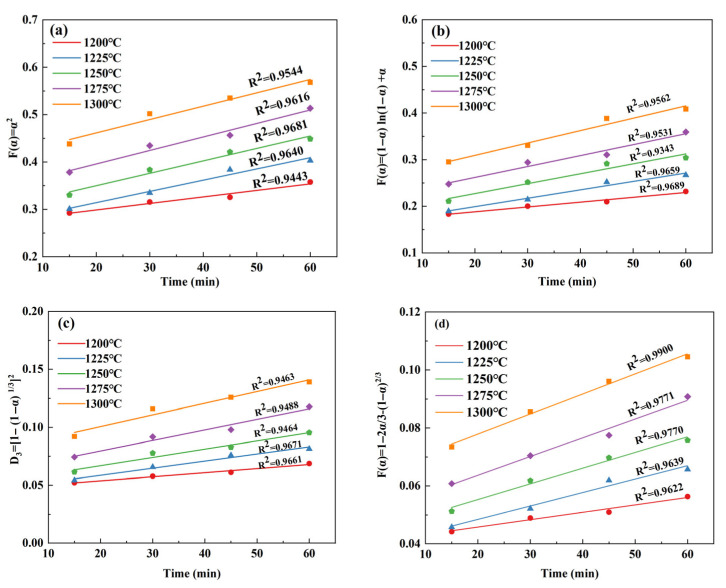
Fitted curves of *α* and *t* for C_4_A_3_$\bar{S}$ at different reaction models: (**a**) flat model; (**b**) cylindrical model; (**c**) spherical model (Jander); and (**d**) spherical model (Ginstling).

By substituting the conversion rate into the above kinetic equation and fitting the relationship between *α* and *t*, it can be clearly seen that the diffusion-controlled kinetic equation provides a more accurate description of the C_4_A_3_$\bar{S}$ formation. The reason for this might lie in the effect of Ca^+^ diffusion on the process of mineral formation. The fitting relationship between *α* and *t* of C_4_A_3_$\bar{S}$ under different reaction models is shown in Figure 5. In Table 5, D_4_ is the most suitable kinetic equation; as demonstrated in Figure 5d, it provides a relatively good quantitative explanation for the formation of C_4_A_3_$\bar{S}$ under different calcination systems. Correspondingly, the sphere model (Ginstling) can represent the formation kinetic model of C_4_A_3_$\bar{S}$ [32]. This conclusion is consistent with Geng’s study on the preparation of C_4_A_3_$\bar{S}$ from solid waste [24], but different from Ma’s study [22], possibly because Ma’s study was based on pure chemical reagents.

#### 3.2.3. Kinetic Parameters of C_4_A_3_$\bar{S}$

Activation energy refers to the energy barrier or threshold that needs to be overcome for a certain reaction to occur. The logarithmic equation of the Arrhenius formula can be used to calculate the activation energy required for cement clinker formation, as shown in Equation (4). The specific method is to establish a linear fitted relation for *-LnK* in relation to 1/*T* and calculate the slope of the fitting line.

(4) LnK=LnA−Ea/RT

where *A*—prefactor; *K*—reaction rate constant (s^−1^); *R*—ideal gas constant (8.314 × 10^−3^ kJ/mol); *Ea*—activation energy (kJ/mol); and *T*—reaction temperature (°C).

The reaction rate constants of C_4_A_3_$\bar{S}$ at different calcination temperatures are shown in Table 6, and they are the slopes of the respective lines in Figure 5. From Table 6, it can be seen that as the temperature continues to increase, the reaction rate constant shows an increasing trend.

**Figure 6:**
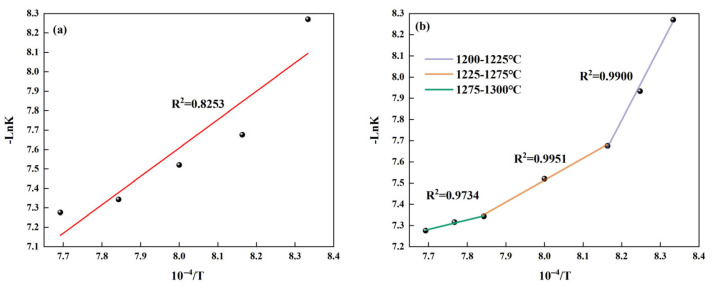
The fitting relationship between *-LnK* and 1/*T* during the formation process of C_4_A_3_$\bar{S}$: (**a**) fitting relationship at 1200–1300 °C and (**b**) fitting relationship for different temperature ranges.

Based on the data in Table 6, the linear fitted relation between *-LnK* and 1/*T* was established as shown in Figure 6a. As shown in this figure, the value of R^2^ is relatively low; it is not reasonable to directly use the formula to calculate the activation energy. Therefore, the temperature range should be divided into three sections, and their activation energies should be calculated separately. In addition, due to the limited number of data points in the ranges of 1200–1225 °C and 1275–1300 °C, we have added the *-LnK* values corresponding to the intermediate temperature points and made a fitting curve as shown in Figure 6b, and the results are shown in Table 7. From this table, it can be seen that as the temperature increases, the activation energy required for C_4_A_3_$\bar{S}$ formation continuously decreases and is significantly lower than that (198.01 kJ/mol, 234 kJ/mol) when Ma and Li prepared C_4_A_3_$\bar{S}$ using pure chemical reagents [22,23]. This result indicates that our study demonstrates significant advantages in the field of low-energy preparation.

### 3.3. High-Temperature Reaction Kinetics of β-C_2_S

#### 3.3.1. Conversion Rate of β-C_2_S

As shown in Table 3, the content of β-C_2_S also increases with the increase in calcination temperature and the prolongation of holding time. During the reaction process, due to the relatively low formation temperature of β-C_2_S, it has not yet reached the level of liquid phase formation. Therefore, its formation process can be regarded as a solid-state reaction, and its formation process is shown in Equation (5).

(5) $$2CaO+SiO_{2}\to 2C_{2}S$$

In this study, the designed content of β-C_2_S is 45%. By using Equation (1) and the data in Table 3, the conversion rates of β-C_2_S under different calcination systems can be obtained, and the results are listed in Table 8.

#### 3.3.2. Kinetic Model of the β-C_2_S Formation

**Figure 7:**
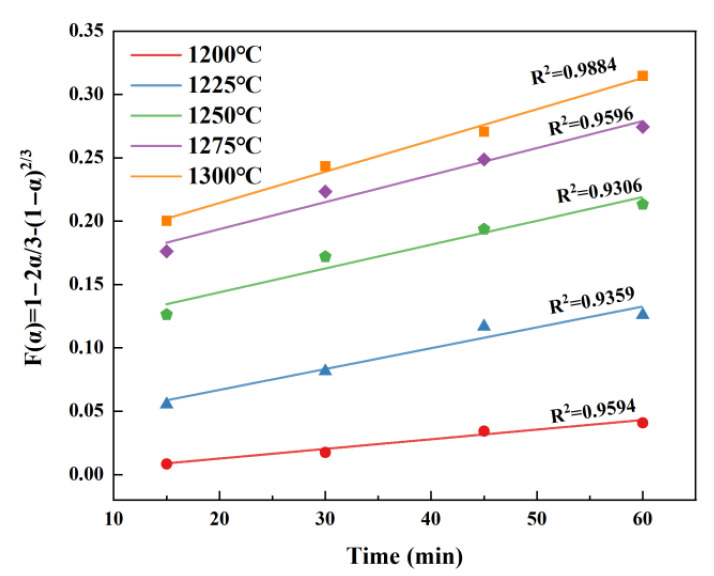
Fitted curves of *α* and *t* for β-C_2_S at different temperatures.

It is similar to that of C_4_A_3_$\bar{S}$. By substituting the conversion rate into the above kinetic equation and fitting the relationship between *α* and *t*, it can be clearly seen that the diffusion-controlled kinetic equation provides a more accurate description of the β-C_2_S formation, as shown in Figure 7. This finding aligns with the study findings of Xu [25] and Hao [26] regarding β-C_2_S.

#### 3.3.3. Kinetic Parameters of β-C_2_S

The reaction rate constants of β-C_2_S at different calcination temperatures are shown in Table 9, and they are the slopes of the respective lines in Figure 7. From this table, it can be seen that as the temperature continues to increase, the reaction rate constant also shows an increasing trend.

**Figure 8:**
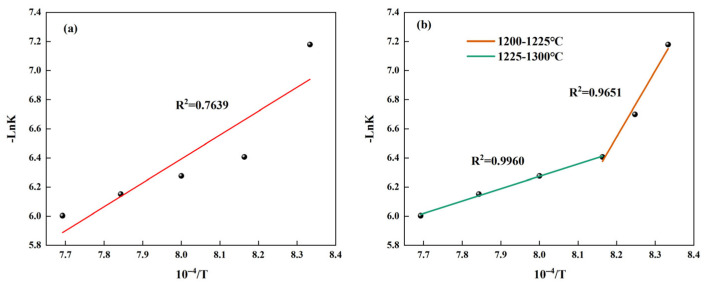
The fitting relationship between *-LnK* and 1/*T* during the formation process of β-C_2_S: (**a**) fitting relationship at 1200–1300 °C and (**b**) fitting relationship for different temperature ranges.

Based on the data in Table 9, the linear fitted relation between *-LnK* and 1/*T* was established as shown in Figure 8a. As shown in this figure, the value of R^2^ is relatively low; it is not reasonable to directly use the formula to calculate the activation energy. Therefore, the temperature range should be divided into two sections, and their activation energies should be calculated separately. In addition, due to the limited number of data points in the range of 1200–1225 °C, we have added the *-LnK* values corresponding to the intermediate temperature points and made a fitting curve as shown in Figure 8b. The results are shown in Table 10. From this table, it can be seen that the activation energy is higher when the temperature range is 1200–1225 °C. This is because the relatively low temperature results in relatively small molecular kinetic energy, thus requiring a higher activation energy. When the temperature gradually increases to the range of 1225–1300 °C, its activation energy is significantly lower than the activation energy (121.5 kJ/mol) obtained by Hao using numerical simulation [26]. This demonstrates that our study holds an edge in low-energy preparation methods.

### 3.4. High-Temperature Reaction Kinetics of CaSO_4_

#### 3.4.1. Conversion Rate of CaSO_4_

The consumption rate of CaSO_4_ refers to the degree of CaSO_4_ consumption in clinkers; it can be described by the ratio between content differences in clinker minerals and raw materials, as shown in Equation (6).

(6) $$\alpha '=\frac{w'-Wr}{Wr}\times 100\%$$

where *w*′—actual content of CaSO_4_ in clinker minerals (%); *α*′—consumption rate of CaSO_4_ (%); and *Wr*—content of CaSO_4_ in raw mineral (%).

In this study, the designed content of CaSO_4_ has been determined to be 15%, and the content of CaSO_4_ in the raw materials ranges from 38.69 to 44.15%. By using Equation (6) and the data in Table 3, the consumption rates of CaSO_4_ under different calcination systems can be obtained, and the results are listed in Table 11.

#### 3.4.2. Kinetic Model of the CaSO_4_ Formation

**Figure 9:**
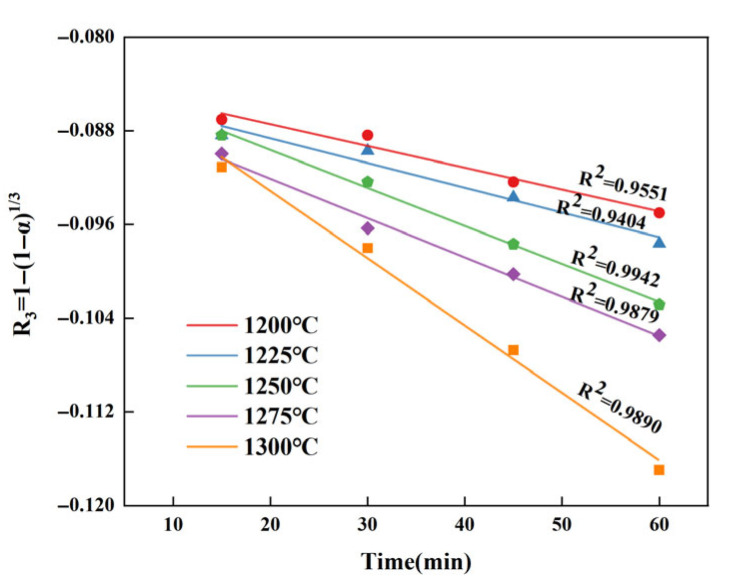
Fitted curves of *α*′ and *t* for CaSO_4_ at different temperatures.

It is similar to that of C_4_A_3_$\bar{S}$ and β-C_2_S. By substituting the data from Table 11 into the kinetic equation in Table 5 for validation, it is not difficult to find that the R_3_ equation under interfacial chemical reactions control can better reflect the consumption of CaSO_4_, as shown in Figure 9.

#### 3.4.3. Kinetic Parameters of CaSO_4_

The reaction rate constants of CaSO_4_ at different calcination temperatures are shown in Table 12, and they are the slopes of the respective lines in Figure 9. From this table, it can be seen that as the temperature continues to increase, the reaction rate constant also shows an increasing trend.

## 4. Conclusions

In order to further elucidate the calcination process of the new solid waste-based HBSAC clinker, this article starts from the perspective of high-temperature reaction kinetics and uses QXRD quantitative analysis and numerical fitting analysis methods. The reaction laws of C_4_A_3_$\bar{S}$, β-C_2_S, and CaSO_4_ were explored, a high-temperature kinetic model of the minerals was established, and the kinetic parameters of each mineral were obtained. This study provides a certain reference for the development of low-carbon cement, and its main conclusions are as follows:

(1) For NHBASC, the C_4_A_3_$\bar{S}$ and β-C_2_S content increases with prolonged holding times and higher calcination temperatures, while CaSO_4_ continues to be consumed. Within the temperature range of 1200–1300 °C, the conversion rates of C_4_A_3_$\bar{S}$ and β-C_2_S both increase with the increase in calcination temperature and the prolongation of holding time, but the conversion rate of β-C_2_S decreases when the temperature is too high. Overall, the conversion rates of β-C_2_S are higher than that of C_4_A_3_$\bar{S}$, indicating that β-C_2_S reacts more thoroughly within this temperature range. The above mineral reaction laws provide a certain basis for optimizing the calcination system, enabling precise design of the clinker mineral composition through the synergistic adjustment of calcination temperature and holding time.
(2) The formation of C_4_A_3_$\bar{S}$ and β-C_2_S is influenced by diffusion mechanisms, and they both satisfy the Glinstling equation. The activation energy required for mineral formation varies in different temperature ranges. The activation energies required to form C_4_A_3_$\bar{S}$ at 1200–1225 °C, 1225–1275 °C, and 1275–1300 °C are 166.28 kJ/mol, 83.14 kJ/mol, and 36.58 kJ/mol, respectively. The activation energies required to form β-C_2_S at 1200–1225 °C and 1225–1300 °C are 374.13 kJ/mol and 66.51 kJ/mol, respectively. Overall, the activation energy required for mineral formation is relatively low, indicating that solid waste-based NHBSAC embodies the advantages of low carbon and energy saving, which meets the development needs of green building materials.
(3) The consumption of CaSO_4_ is controlled by the interfacial chemical reaction mechanism, satisfying the R_3_ equation. By obtaining the reaction mechanism and kinetic parameters of CaSO_4_, it can be known that the reaction rates of CaSO_4_ increase with the increase in calcination temperature and the extension of holding time. Therefore, in the process of clinker preparation, the holding time should not be too long to avoid affecting the residual CaSO_4_ content in NHBSAC.

This study mainly conducted theoretical exploration on the formation laws and kinetic reaction parameters of minerals. The next step will use the above theoretical results to guide the optimization and matching of mineral composition, prepare corresponding clinker, and conduct actual properties testing and analysis on the obtained clinker.

## Figures and Tables

**Table 1:** Main chemical composition of raw materials.

Raw Material	CaO/%	Al_2_O_3_/%	SiO_2_/%	Fe_2_O_3_/%	SO_3_/%	MgO/%	TiO_2_/%	Others/%	LOI/%
PCA	62.36	0.45	0.85	0.24	26.09	1.07	0.00	0.36	8.58
FA	3.44	30.37	52.17	5.64	0.79	0.73	1.22	3.24	2.40
CS	67.75	1.10	2.18	0.17	1.69	0.10	0.00	2.08	24.93
BX	1.07	75.09	14.83	1.53	0.18	0.48	4.50	1.62	0.70

Note: LOI refers to loss on ignition.

**Table 2:** Experimental schemes for each group and the proportion of raw materials.

No.	Calcination Temperature/°C -Holding Time/min	Raw Material/%			
		PCA	FA	CS	BX
1 #	1200-15	45.55	16.78	28.36	9.31
2 #	1200-30	44.17	17.39	27.83	10.61
3 #	1200-45	43.92	17.50	27.21	11.37
4 #	1200-60	43.74	17.64	26.54	12.08
5 #	1225-15	44.66	16.95	28.90	9.49
6 #	1225-30	44.41	17.21	27.71	10.67
7 #	1225-45	44.27	17.36	26.91	11.46
8 #	1225-60	44.30	17.41	26.28	12.01
9 #	1250-15	44.87	16.81	28.91	9.41
10 #	1250-30	44.82	16.98	27.67	10.53
11 #	1250-45	44.97	17.04	26.58	11.41
12 #	1250-60	45.24	17.01	25.85	11.90
13 #	1275-15	47.58	16.13	27.10	9.19
14 #	1275-30	47.41	16.18	25.89	10.52
15 #	1275-45	47.32	16.26	25.04	11.38
16 #	1275-60	47.32	16.26	24.39	12.03
17 #	1300-15	49.92	15.55	25.51	9.02
18 #	1300-30	49.61	15.60	24.49	10.30
19 #	1300-45	49.46	15.55	23.64	11.35
20 #	1300-60	49.14	15.55	23.17	12.13

**Table 3:** Actual content of clinker minerals obtained by internal standard method analysis.

NO.	Actual Content of Clinker Minerals/%				NO.	Actual Content of Clinker Minerals/%			
	$C_{4}A_{3}\bar{S}$	β-C_2_S	CaSO_4_	$C_{5}S_{2}\bar{S}$		$C_{4}A_{3}\bar{S}$	β-C_2_S	CaSO_4_	$C_{5}S_{2}\bar{S}$
1 #	18.92	12.22	14.35	18.39	11 #	22.71	43.72	13.59	--
2 #	19.66	17.13	14.25	19.53	12 #	23.44	44.67	13.21	--
3 #	20.06	23.04	13.97	21.96	13 #	21.53	42.10	14.06	--
4 #	20.93	24.78	13.78	15.23	14 #	22.80	44.68	13.68	--
5 #	19.19	28.10	14.25	10.32	15 #	23.65	45.68	13.40	--
6 #	20.25	32.63	14.16	7.17	16 #	25.08	46.46	13.02	--
7 #	21.69	37.10	13.87	5.79	17 #	23.17	40.20	14.06	--
8 #	22.22	38.03	13.59	3.55	18 #	24.55	41.21	13.78	--
9 #	20.11	38.07	14.25	--	19 #	25.60	42.39	12.92	--
10 #	21.67	41.82	13.97	--	20 #	26.38	43.77	12.16	--

Note: The data of each group in this table are the average results of three experiments, which ensure the accuracy and stability of the experimental results.

**Table 4:** Conversion rates of C_4_A_3_$\bar{S}$ under different calcination systems (%).

Calcination Temperature/°C	Holding Time/min			
	15	30	45	60
1200	54.06	56.17	57.32	59.81
1225	54.84	57.87	61.96	63.49
1250	57.45	61.92	64.89	66.98
1275	61.51	65.15	67.56	71.66
1300	66.19	70.13	73.14	75.37

**Table 5:** Control mechanisms and kinetic equations of solid-state reaction [30,31].

Equation Number	Equation	Model	Control Mechanism
D_1_	$\alpha^{2}=Kt$	Flat model	Diffusion control
D_2_	(1−α)ln(1−α)+α=Kt	Cylindrical model	
D_3_	${\left[1-{\left(1-\alpha \right)}^{1/3}\right]}^{2}=Kt$	Spherical model (Jander)	
D_4_	$1-2\alpha /3-{\left(1-\alpha \right)}^{2/3}=Kt$	Spherical model (Ginstling)	
R_1_	−ln(1−α)=Kt	Spherical model (first-order reaction)	Interface chemical reaction control
R_2_	$1-{\left(1-\alpha \right)}^{1/2}=Kt$	Cylindrical model	
R_3_	$1-{\left(1-\alpha \right)}^{1/3}=Kt$	Spherical model (zero-order reaction)	
R_4_	α=Kt	Flat model	
A_1_	${\left[-ln(1-\alpha )\right]}^{1/4}=Kt$	--	Nucleation growth control
A_2_	${\left[-ln(1-\alpha )\right]}^{1/3}=Kt$	--	
A_3_	$[-ln(1-\alpha ){]}^{1/2}=Kt$	--	

**Table 6:** Reaction rate constants of C_4_A_3_$\bar{S}$ under various temperatures.

Equation	Temperature/°C	*K*
$D_{4}=1-2\alpha /3-{\left(1-\alpha \right)}^{2/3}=Kt$	1200	2.56 × 10^−4^
	1225	4.63 × 10^−4^
	1250	5.41 × 10^−4^
	1275	6.47 × 10^−4^
	1300	6.92 × 10^−4^

**Table 7:** Activation energy of C_4_A_3_$\bar{S}$ formation in different temperature ranges.

**Temperature Ranges/**°**C**	1200–1225	1225–1275	1275–1300
**Activation Energy/kJ/mol**	166.28	83.14	36.58

**Table 8:** Conversion rates of β-C_2_S under different calcination systems (%).

Calcination Temperature/°C	Holding Time/min			
	15	30	45	60
1200	25.79	36.16	48.63	52.31
1225	59.33	68.89	78.32	80.28
1250	80.36	88.28	92.30	94.30
1275	88.87	94.32	96.43	98.07
1300	84.87	87.00	89.48	92.40

**Table 9:** Reaction rate constants of β-C_2_S under various temperatures.

Equation	Temperature/°C	*K*
$D_{4}=1-2\alpha /3-{\left(1-\alpha \right)}^{2/3}=Kt$	1200	7.62 × 10^−4^
	1225	1.65 × 10^−3^
	1250	1.88 × 10^−3^
	1275	2.13 × 10^−3^
	1300	2.47 × 10^−3^

**Table 10:** Activation energy of β-C_2_S formation in different temperature ranges.

**Temperature Ranges/°C**	1200–1225	1225–1275
**Activation Energy/kJ/mol**	374.13	66.51

**Table 11:** Consumption rates of CaSO_4_ under different calcination systems (%).

Calcination Temperature/°C	Holding Time/min			
	15	30	45	60
1200	−28.45	−28.93	−30.35	−31.30
1225	−28.93	−29.40	−30.82	−32.24
1250	−28.93	−30.35	−32.26	−34.14
1275	−29.88	−31.77	−33.19	−35.09
1300	−29.90	−31.30	−35.56	−39.35

Note: “−” represents consumption.

**Table 12:** Reaction rate constants of CaSO_4_ under various temperatures.

Equation	Temperature/°C	*K*
$R_{3}=1-{\left(1-\alpha \right)}^{1/3}=Kt$	1200	−1.86 × 10^−4^
	1225	−2.11 × 10^−4^
	1250	−3.25 × 10^−4^
	1275	−3.36 × 10^−4^
	1300	−5.75 × 10^−4^

## Data Availability

The raw data supporting the conclusions of this article will be made available by the authors on request.

## Abbreviations

The following abbreviations are used in this manuscript:

Abbreviation	Chemical Formula	Chemical Name	Mineral Name
$C_{5}S_{2}\bar{S}$	4CaO·2SiO_2_·CaSO_4_	Calcium Sulfosilicate	Ternesite
$C_{4}A_{3}\bar{S}$	3CaO·3Al_2_O_3_·CaSO_4_	Calcium Sulphoaluminate	Ye’elimite
C_4_AF	4CaO·Al_2_O_3_·Fe_2_O_3_	Tetracalcium Aluminoferrite	Brownmillerite
β-C_2_S	2CaO·SiO_2_	Dicalcium Silicate	Belite
